# Masking level differences under two different measurement conditions: A normative study of young adults

**DOI:** 10.1002/brb3.70011

**Published:** 2024-09-18

**Authors:** Sule Cekic, Banu Mujdeci, Kursad Karakoc, Banu Bas

**Affiliations:** ^1^ Faculty of Health Sciences, Audiology Department Ankara Yildirim Beyazit University Ankara Turkey

**Keywords:** auditory perception, binaural interaction, hearing

## Abstract

**Objective:**

The objective of this study was to determine the gender‐specific normative values of masking level difference (MLD) in healthy young adults for two different measurement conditions.

**Methods:**

One hundred young adults between the ages of 19 and 25 were included. Tympanometry, pure tone audiometry, and MLD were performed. In the first MLD measurement condition, the threshold level where the signal was out of phase and the noise was in phase (SπNo) was subtracted from the threshold level where the signal and noise were in phase (SoNo). In the second MLD measurement condition, the threshold level where the signal was in phase and the noise was out of phase (SoNπ) was subtracted from the threshold level where the signal and noise were in phase (SoNo). The mean test scores were obtained in decibels. Comparisons were made in terms of gender and conditions.

**Results:**

The mean MLD for SoNo‐SπNo condition was 10.3 ± 1.99 dB. For SoNo‐SoNπ condition, the mean MLD was 6.72 ± 2.38 dB. A significant difference was determined between the MLD under two different measurement conditions (*p* <.05). There was no significant difference in terms of gender (*p* >.05).

**Conclusion:**

Mean normative values of MLD test scores in gender‐specific healthy young adults for two different measurement conditions are presented.

## Graphical abstract

This study provides the mean (and standard deviation) MLD scores which are obtained under “SoNo‐SπNo” and “SoNo‐SoNπ” measurement conditions in healthy young adults by manually applied adaptive procedure method and contributes to normative values for both genders.

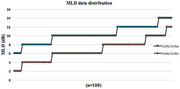

## 1 INTRODUCTION

To be able to hear and understand acoustic stimuli properly, auditory processes and auditory mechanisms such as localization, lateralization, auditory discrimination, recognition of temporal aspects of auditory stimuli (decoding, masking, integration, and sequencing), and hearing in the presence of impaired acoustic signals are substantial. Furthermore, binaural hearing provides significant advantages on auditory mechanisms and functions which can be evaluated with tests using both verbal and non‐verbal stimuli (American Speech Language Hearing Association, 2021). Binaural interaction describes the inter‐aural relations of the signal or/and the noise presented simultaneously to both ears. The signal detection threshold sensitivity improves in the listening conditions in which there is a phase difference between the signal and the noise compared to the conditions in which both the signal and the noise are in‐phase (Lynn et al., 1981). Masking level difference (MLD) is a binaural interaction phenomenon that describes the improvement in the detection threshold due to phase differences of simultaneously presented signal and noise during the presentation (de Carvalho et al., 2021). This improvement in function is the result of a release from masking (Olsen et al., 1976) and occurring at the level where information from the two ears is first integrated (Davies, 2016). Wilson et al. (2003) describe the two conditions—homophasic and antiphasic—in their study of MLD test protocol for clinic use as follows: The homophasic condition implies that both the signal and the noise are in‐phase at the ears, and in phase signal‐to‐noise is denoted by the symbols “SoNo” or “SπNπ” in π radians (180°). In the antiphasic condition, there is a phase difference of π radians (180°) between the signal and the noise and is indicated by the symbols “SπNo” or “SoNπ.” In this second condition, the waveforms presented to the ears differ in terms of both temporal and amplitude characteristics. The MLD, which is obtained by subtracting the thresholds in those different listening conditions, primarily evaluates brainstem functions and is sensitive even in non‐severe brainstem dysfunctions (Noffsinger et al., 1984; Olsen & Noffsinger, 1976).

It is known for decades that gender differences in auditory physiology (Nolan, 2020) are lifelong phenomena, and it is advised to compare a patient's hearing data to a large sample of his/her healthy age‐cohort in their community to detect and treat abnormal declines in hearing (O'Connor, 1990). In other words, age and gender (Deeks et al., 2009) even race and ethnicity (Farina et al., 2023) are important variables in the field of health research, and they often have a significant effect on the analyzed parameters. As a matter of fact, most of the hearing‐related evaluation tools are interpreted using normative data specific to gender and different age groups (and sometimes race and ethnicity). Regarding the MLD test, no gender‐specific study has been found in young adults in the Turkish population. However, knowing the gender‐specific MLD scores in different age groups is a fundamental requirement for effective applications. Furthermore, different antiphasic conditions (“SπNo” or “SoNπ”) in MLD calculations may result in different scores. So, this study aimed to determine the mean (and standard deviation‐SD) MLD scores which are obtained under “SoNo‐SπNo” and “SoNo‐SoNπ” measurement conditions in healthy young adults by the manually applied adaptive procedure method and to contribute to the establishment of normative values for both genders.

## 2 MATERIALS AND METHODS

### 2.1 Participants

This study was carried out in Ankara Yildirim Beyazit University Audiology Laboratories. Ethics committee approval was obtained from Ankara Yildirim Beyazit University Ethics Committee (27.12.2019/47). The purpose of the study was explained to the participants, and their written consent was obtained.

A total of 100 individuals between the ages of 19 and 25 were included in the study. The inclusion criteria of all participants in the study were to be in the age range of 19–25 to have bilateral air‐conduction pure‐tone hearing threshold ≤25 dB HL between the frequencies of 125 and 8000 Hz and bone conduction pure‐tone hearing thresholds ≤25 dB HL between the frequencies of 500 and 4000 Hz to have a bilateral type A tympanogram and to have bilateral ipsilateral and contralateral acoustic reflexes. Individuals with an outer ear, middle ear, or inner ear pathology; previous ear surgery; history of acoustic trauma; and complaints regarding central auditory processing were not included in the study.

## 3 METHODS

Otoscopic ear examination was performed on individuals who agreed to participate in the study. Air conduction and bone conduction hearing thresholds of all participants were determined in the acoustic booth (Otatech, Turkey) using a clinical audiometer (Madsen Astera 2; Otometrics). Pure‐tone air conduction hearing thresholds were determined between 125 and 8000 Hz with supra‐aural headphones (TDH 39P), and pure‐tone bone conduction hearing thresholds were determined between 500 and 4000 Hz with bone conduction vibrator (Radioear B71). Tympanometry (Madsen OTOflex 100; Otometrics) using a 226 Hz probe tone and ipsilateral and contralateral acoustic reflex threshold measurements in the 500–4000 Hz range were performed. As a result of these measurements, the MLD test was performed on the individuals who met the inclusion criteria by switching to the MLD test screen in the clinical audiometer (Madsen Astera 2; Otometrics) software.

For the masking level difference test, participants were given the following instruction: “You will hear a continuous noise in both ears. When you are constantly hearing the sound of noise, you will also hear a signal tone in both ears from time to time. Press the button every time you hear the signal tone, regardless of the noise.” After the instruction, 500 Hz was selected as the frequency on the MLD test screen. Narrowband noise at 50 dB HL was presented binaurally and continuously. The thresholds in homophasic (SoNo) and anti‐phasic (SπNo, SoNπ) conditions were determined by setting the initial intensity of the signal tone at 70 dB HL and using the “descending method” with 2 dB steps. The level at which the participant heard the signal at a 50% rate was set as the threshold (McPherson et al., 2011).

Masking level difference test scores were obtained for two different measurement conditions. In the first measurement condition, to determine the MLD test score, the threshold level where the signal was out of phase and the noise was in phase (SπNo) was subtracted from the threshold level where the signal and noise were in phase (SoNo). In the second measurement condition, to determine the MLD test score, the threshold level where the signal was in phase and the noise was out of phase (SoNπ) was subtracted from the threshold level where the signal and noise were in phase (SoNo). The mean test scores (and standard deviation values) were obtained in decibels. Measurement formulas for MLD are presented as follows:

$$\begin{array}{ccc} & & \mathrm{MLD}\;\mathrm{test}\;\mathrm{score}\;(\mathrm{dB})\;\mathrm{in}\;\mathrm{the}\;\mathrm{first}\;\mathrm{condition}:\\ & & \mathrm{SoNo}\;\mathrm{threshold}\;\mathrm{level}\;\left(\mathrm{dB}\;\mathrm{HL}\right)-S\pi \mathrm{No}\;\mathrm{threshold}\;\mathrm{level}\;\left(\mathrm{dB}\;\mathrm{HL}\right) \end{array}$$

$$\begin{array}{ccc} & & \mathrm{MLD}\;\mathrm{test}\;\mathrm{score}\left(\mathrm{dB}\right)\mathrm{in}\;\mathrm{the}\;\mathrm{second}\;\mathrm{condition}\;:\\ & & \mathrm{SoNo}\;\mathrm{threshold}\;\mathrm{level}\;(\mathrm{dB}\;\mathrm{HL})-\mathrm{SoN}\pi \;\mathrm{threshold}\;\mathrm{level}\;(\mathrm{dB}\;\mathrm{HL}) \end{array}$$

### 3.1 Statistical analysis

Statistical Package for the Social Sciences (SPSS‐version 24) was used for statistical analysis of the data. The normal distribution of the data was analyzed using Shapiro Wilk and Kolmogorov Smirnov tests. Quantitative data were given as mean, standard deviation, median, minimum, and maximum, and qualitative data were given as frequency (percentage). Wilcoxon signed‐rank test was used to compare the MLD test scores under two different situations, and the Mann–Whitney *U* test was used to compare the MLD test scores under two different situations according to gender. The significance level was accepted as *p* <.05.

## 4 RESULTS

The mean age of the 100 individuals included in the study was 20.99 ± 1.41 years. The gender and age information of the individuals is shown in Table 1.

**TABLE 1:** Distribution of gender and age averages of individuals.

Gender	*n*	Age (year)	
		Mean ± standard deviation	Min.–max.
Female	80	20.98 ± 1.34	19.0–25.0
Male	20	21.00 ± 1.68	19.0–25.0
Total	100	20.99 ± 1.41	19.0–25.0

For the first MLD test score, the threshold level where the signal was out of phase (Sπ) and the noise was in phase (No) (SπNo) was subtracted from the threshold level where the signal and noise were in phase (SoNo). In this case, the mean MLD test score was 10.3 dB (SD: 1.99). For the second MLD test score, the threshold level where the signal was in phase (So) and the noise (Nπ) was out of phase (SoNπ) was subtracted from the threshold level where the signal and noise were in phase (SoNo). In this case, the mean MLD test score was 6.72 dB (SD: 2.38).

Masking level difference test scores obtained under two different situations are given in Table 2.

**TABLE 2:** Comparison of masking level difference (MLD) test scores obtained under two different situations.

	SoNo‐SπNo (*n* = 100) median (min.–max.)	SoNo‐SoNπ (*n* = 100) median (min.–max.)	*p* ^a^
MLD test score (dB)	10.3 (6–14)	6.72 (2–12)	.000

^a^ Wilcoxon signed‐ranks test.

A significant difference was determined between the MLD test scores under two different measurement conditions (*p* <.05).

Masking level difference test scores obtained under two different measurement conditions according to gender are given in Table 3.

**TABLE 3:** Comparison of masking level difference (MLD) test scores obtained under two different measurement conditions by gender.

MLD test score (dB)	Gender		*p^a^*
	Male (*n* = 20)	Female (*n* = 80)	
SoNo‐SπNo median (min.–max.)	11.0 (6–14)	10.12 (6–14)	.086
SoNo‐SoNπ median (min.–max.)	7.0 (2–12)	6.65 (2–12)	.686

^a^ Mann–Whitney *U* test.

There was no significant difference between the MLD test scores by gender under two different measurement conditions (*p* >.05).

**FIGURE 1:**
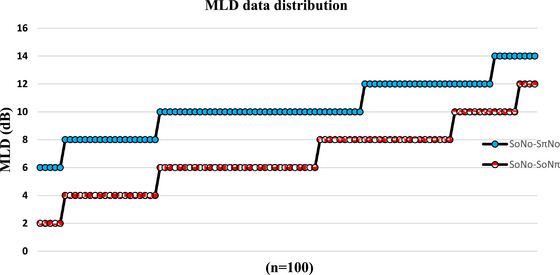
Sorting the masking level difference (MLD) data of the 100 individuals from lowest to highest for test scores under two different measurement conditions (SoNo‐SπNo and SoNo‐SoNπ).

The order of the MLD data of the 100 individuals for the test scores under two different measurement conditions (SoNo‐SπNo and SoNo‐SoNπ) is given in Figure 1, from lowest to highest.

## 5 DISCUSSION

In this study, gender‐specific normative data on MLD in Turkish young adults were provided. The mean (and SD) values of MLD test scores under “SoNo‐SπNo” and “SoNo‐SoNπ” measurement conditions were determined as 10.3 dB (SD: 1.99) and 6.72 dB (SD: 2.38), respectively. Since the publication of the early studies on MLD in the 1948s and 1949s, there has been a steady increase in the knowledge of binaural masking phenomena (Egan et al., 1969). The characteristic MLD calculation paradigm includes homophasic and antiphasic masking conditions (Licklider, 1948). It has been noted that it is easier to hear the signal under the antiphasic (SπNo) condition than under the homophasic (SoNo or SπNπ) condition, due to the signal coming from the center of the head in the homophasic condition and the presence of additional timing and intensity cues in the antiphasic condition (McPherson et al., 2011). The SπNπ condition is a special condition in which the temporal properties of the waveforms presented to the ears are preserved, but the amplitude properties are reversed. The SπNπ condition produces the same thresholds as the SoNo condition (Egan, 1965; Sever & Small, 1979). Indeed, in this study, in the MLD test scores obtained under two different measurement conditions in healthy young adults, a higher value was observed in the first measurement condition obtained by subtracting the threshold level in the SπNo (antiphasic) condition from the threshold level in the SoNo (homophasic) condition. This finding suggests that hearing in the SπNo (antiphasic) condition is easier in healthy young adults. Wilson et al. (2003) conducted serial trials to develop a simple application protocol for MLD testing at 500 Hz that could be used in clinical practice. They reported that 95% of the participants achieved a test score of 10 dB or more and that this value was the reference value for normality. In general, normal MLD scores are reported at values ranging from 10.6 to 11.7 dB (Harris et al., 1992; Jerger et al., 1984; Olsen et al., 1976), as in more recent studies (do Couto Mendes et al., 2017; Wilson et al., 2003) mostly values of 10 dB or greater for normative data. Similarly, the mean MLD test score of Turkish young adults was determined as 10.3 dB for the “SoNo‐SπNo” measurement condition in our study, and this value is consistent with other normative data in the literature (Guven & Mutlu, 2003). Guven and Mutlu (2003), for example, obtained a mean MLD score of 10.92 dB in a study of the Turkish population in which only SoNo‐SπNo status was examined. However, in our study, we also analyzed the “SoNo‐SoNπ ” measuring condition and came up with a value of 6.72 dB, which was much lower than the other measurement condition and was consistent with the literature (Clinard et al., 2017). The difference in these two measurement conditions is due to differences in signal combinations. On the other hand, the reason for the different normative data in the literature is thought to be due to differences in the test procedure such as differences in noise and signal combinations, differences in masking noise type, length of the signal, and different calibrations between the audiometers in which the test is performed.

Although there is an infinite number of phase relationships for signals or noises between the ears, the SπNo condition is typically used in audiology to describe the antiphasic condition. In practice, in order to obtain the MLD, it is suggested to subtract the threshold in the SπNo condition from the threshold in the SoNo condition (Wilson et al., 2003). However, it is not unusual to see SoNπ condition in MLD calculations instead of SπNo (Clinard et al., 2017). The value obtained by subtracting the threshold in the SoNo condition from the threshold in the SoNπ condition is usually 2–3 dB lower than the value obtained from the reference practice mentioned above (Egan, 1965; Olsen et al., 1976; Schoeny & Carhart, 1971). Another study in the literature reported that the MLD value obtained in the SoNo‐SπNo measurement condition is ∼2 dB larger than the value obtained in the SoNo‐SoNπ measurement condition (Wightman, 1971). Likewise, the study of Aithal et al. (2006) emphasized that the average MLD value in the SπNo condition is significantly higher than the average MLD value in the SoNπ condition. In our study, the MLD test score for Turkish young adults was found to be 10.3 dB in the first measurement condition (SoNo‐SπNo), and 6.72 dB in the second measurement condition (SoNo‐SoNπ). The difference between the two measurement conditions was 3.58 dB, which is considered to be compatible with the values in the literature. A study in the literature discussing the reason for the difference between the two conditions noted that for the SoNπ condition, mask noise information could be combined with the matching contralateral signal information in the left insula lobe with the suppression effect when passing from the right hemisphere to the left hemisphere through the corpus callosum. Thus, the signal detection threshold for the SoNπ condition increases, and the MLD score for the SoNπ condition decreases compared to the SπNo condition (Wack et al., 2012).

In our study, the comparison of the MLD test scores by gender exhibited no significant difference between men and women for MLD values obtained under two different conditions. As a matter of fact, it has been reported that there is no statistically significant difference in the MLD values between men and women in normative studies (de Paula et al., 2012; Martins et al., 2018).

Binaural hearing provides considerable benefits in hearing sensitivity, loudness perception, general speech perception, and speech perception in adverse conditions (Musiek & Chermak, 2013). It is presented that the binaural interaction tests, as a part of a test battery, can be used in behavioral assessment of auditory processing skills (American Speech Language Hearing Association, 2021). We think that this study, which was conducted to improve the normative data of the MLD test and which evaluates the binaural interaction mechanism, contributes to the current literature.

Furthermore, with the use of different signal presentation conditions, the MLD test can be used in diagnosis of central auditory processing disorder, differential diagnosis of hearing, monitoring hearing health, and auditory rehabilitation. It is mentioned in the previous paragraphs that test results may vary under different presentation conditions in MLD test. Reasons for this are that SoNo and SoNπ conditions may force different registration of the auditory system. For example, SoNo primarily tests spatial auditory processing ability, while SoNπ can evaluate the ability of integrating complex auditory signals. Furthermore, differences in processing capacities, processing pathologies, or the condition of the individuals may affect spatial hearing to that extent and may bring different results under different presentation conditions for the same person.

The auditory system processes subtle inter‐aural difference cues of time and intensity (Hall et al., 2004) by inter‐aural interaction. One of the tests in which binaural interaction is evaluated through the behavioral procedure is the MLD. The mid‐brainstem level is thought to be responsible for MLD in general (Musiek & Chermak, 2013). In this sense, it is assumed that the MLD test is sensitive to brainstem pathologies and may have results affected by some brain lesions (Kramer et al., 1987; Lynn et al., 1981). It is also stated that the MLD test can be applied to detect lesions in the brainstem and cortical structures, including the corpus callosum, depending on the extent of the pathology (Bellis, 2011). This study was conducted to determine normative data for Turkish young adults. We anticipate that it will contribute to the process of using the MLD test in brainstem pathologies and brain lesions in future studies.

The age group in this study was determined to include young adults. Although this may seem like a limitation at first, it would be advantageous to provide normative data for individuals in a narrow age range. As a matter of fact, it is seen in the literature that MLD studies are often designed to include individuals in a certain age range, as in our study. The developmental effects in MLD for children aged between 5 and 10 (Hall et al., 2004), MLD in school aged children (between age of 7 and 12) (de Carvalho et al., 2021), and women aged between 20 and 30 (do Couto Mendes et al., 2017) are some examples. On the other hand, age effects in MLD for adults may be studied in the future.

The main limitation of our study is that the selection of the sample was based on the declaration of subjects regarding central auditory processing complaints. However, using some screening assessment of the central auditory processing would provide proof during selection of samples. The main reason for this is that the practical screening tools related to CAP are not easily accessible, and furthermore, we encountered the similar subject selection methods in some literature (Clinard et al., 2017; Hall et al., 2004). Another issue that we think needs to be addressed is the sample size imbalance between groups. In fact, an equal sample size is widely and ideally preferred; however, group inequality is a situation encountered in many studies and may not be a significant indicator of a poor study (Vanhove, 2015). Nevertheless, it would be better if the group sample sizes were close to each other.

## 6 CONCLUSION

The MLD test is an efficient, fast, and easy‐to‐apply test for the evaluation of binaural interaction. In this study, mean (and SD) normative values of MLD test scores in gender‐specific healthy young adults under “SoNo‐SπNo” and “SoNo‐SoNπ” measurement conditions were presented. A significant difference was determined between the MLD test scores under two different measurement conditions; however, there was no significant difference between the MLD test scores by gender under two different measurement conditions. The normative values determined in this study can be used as a reference for individuals in this age group in auditory interaction evaluations. In addition, it will be a reference for normative studies to be conducted in other age groups.

## AUTHOR CONTRIBUTIONS

**Sule Cekic**: Conceptualization; methodology; visualization; supervision; writing—original draft; writing—review and editing; software. **Banu Mujdeci**: Conceptualization; formal analysis; writing—review and editing; methodology; validation. **Kursad Karakoc**: Conceptualization; data curation; writing—original draft; visualization; investigation; resources. **Banu Bas**: Data curation; investigation.

## CONFLICT OF INTEREST STATEMENT

The authors no conflicts of interest.

### PEER REVIEW

The peer review history for this article is available at https://publons.com/publon/10.1002/brb3.70011.

## Data Availability

The data that support the findings of this study are available on request from the corresponding author. The data are not publicly available due to privacy or ethical restrictions.
